# Mothers’ Domestic Responsibilities and Well-Being During the COVID-19 Lockdown: The Moderating Role of Gender Essentialist Beliefs About Parenthood

**DOI:** 10.1007/s11199-022-01307-z

**Published:** 2022-07-05

**Authors:** Kjærsti Thorsteinsen, Elizabeth J. Parks-Stamm, Marie Kvalø, Marte Olsen, Sarah E. Martiny

**Affiliations:** 1 Department of Psychology, Research Group Social Psychology, UiT The Arctic University of Norway, Tromsø, Norway; 2 Department of Psychology, University of Southern Maine, Portland, ME USA

**Keywords:** COVID-19, Well-being, Mothers, Sex roles, Ideology, Childcare, Division of labor

## Abstract

The present work investigates how the increased domestic responsibilities created by the Spring 2020 lockdown of the COVID-19 pandemic in Norway and gender ideologies relate to the well-being of mothers with elementary school children. In June 2020, we conducted a cross-sectional online study including current and retrospective measures with 180 mothers (*M*_age_ = 39.96 years, *SD* = 6.11) of elementary school children across Norway. First, in line with earlier research on the strain of the pandemic on parents, and especially mothers, we found that Norwegian mothers’ well-being during the lockdown significantly declined compared to before the lockdown (both measured retrospectively). Furthermore, mothers’ well-being after the Spring 2020 lockdown did not immediately return to pre-lockdown levels. Finally, we predicted that gender ideologies (i.e., essentialist beliefs about parenthood) would exacerbate the negative impact of increased domestic responsibilities (i.e., childcare and housework) on mothers’ well-being (i.e., *higher standard-higher stress* hypothesis). As predicted, for mothers who more strongly endorsed the belief that mothers are instinctively and innately better caretakers than fathers, perceptions of increased domestic responsibilities were associated with lower well-being post-lockdown. These findings point to the specific challenges mothers face in times of crisis, and the importance of addressing and confronting seemingly benevolent ideologies about motherhood that place additional burdens on women.

The outbreak of the COVID-19 pandemic led to a major health crisis starting in Winter 2019 and continuing through Winter 2021 and Spring 2022. To restrict the spread of the virus, in the Spring and early Summer of 2020, many countries implemented strict and far-reaching policy regulations including the shutdown of major parts of public life. These lockdowns negatively affected people’s mental health and well-being worldwide (e.g., Adams-Prassl et al., 2020; Davillas & Jones, 2020; Mazza et al., 2020; Rajkumar, 2020; Viner et al., 2022). Importantly, evidence suggests that the pandemic and related restrictions affected some groups more than others. One group that experienced dramatic changes in daily life is working parents of school-aged children. With schools and daycare centers closed, established daily routines disappeared and parents had to find new ways to combine childcare (including homeschooling), housework, and paid work (Global Gender Gap Index & World Economic Forum, 2021; Power, 2020). Thus, not surprisingly, research shows negative effects of the pandemic on families with young children (Gassman-Pines et al., 2020; Janssen et al., 2020; Patrick et al., 2020) versus older adults (Sterina et al., 2022) and on the well-being of parents (e.g., Cameron et al., 2020; Cusinato et al., 2020; Etheridge & Spantig, 2020; Harth & Mitte, 2020; Huebener et al., 2021; Racine et al., 2022).

Extending this research, in the present work we investigated the well-being of mothers of elementary school-aged children in Norway before, during, and after the lockdown using a cross-sectional study with current and retrospective data. In addition to looking at the role of domestic responsibilities (i.e., childcare and housework) in mothers’ well-being, we further investigated the role gender ideologies play in mothers’ well-being. Specifically, we predicted that the relation between mothers’ share of childcare and housework and their well-being would be moderated by their essentialist beliefs about parenthood, a type of gender ideology that reflects beliefs about women’s innate and natural role as mothers and being a better caregiver (i.e., gender essentialism; Gaunt, 2006). Investigating the role of gender ideology in relation to the consequences of the pandemic and lockdown for mothers’ well-being is important because well-being is associated with productivity, social relationships, health, and longevity (Diener & Seligman, 2004), and crisis situations have previously been shown to exacerbate existing gender inequalities. In addition, parents’ well-being is important not only for parents themselves but also for their children; research shows a consistent link between parents’ mental health and their children’s outcomes (e.g., Rutter & Quinton, 1984; Smith, 2004).

## Family-Level Variables Affecting Parents’ Well-Being

Emerging research shows that several family-level variables are associated with the decrease in parents’ well-being during the COVID-19 pandemic. Besides the financial situation and related worries (Adams-Prassl et al., 2020), the crisis lowered relative life satisfaction especially for parents with young children (Huebener et al., 2021). In addition, research indicates that women generally showed a stronger decline in life satisfaction and mental health during the crisis than men (Adams-Prassl et al., 2020; de Pedraza et al., 2020; Etheridge & Spantig, 2020; Mazza et al., 2020; Oreffice & Quintana-Domeque, 2020). This is also true for mothers compared to fathers (e.g., Huebener et al., 2021; Möhring et al., 2021). This raises the question of whether gender differences in well-being can be explained by the greater increase in domestic responsibilities (i.e., childcare and housework) that women reported during the crisis in comparison to men (e.g., Andrew et al., 2020; Del Boca et al., 2020; Oreffice & Quintana-Domeque, 2020; but see Carlson et al., 2021, who argue increases in fathers’ contributions to housework and childcare during the lockdown led to a more gender-egalitarian balance).

Interestingly, studies investigating whether gender differences in the lockdown-induced increase in domestic responsibilities drive the gender gap in well-being show inconsistent results. On one hand, several studies have found that the gender gap in well-being is reduced when controlling for time spent on childcare and housework (Escudero-Castillo et al., 2021; Etheridge & Spantig, 2020; Pierce et al., 2020), and that increased family responsibilities due to COVID-19 negatively predicted working women’s (but not working men’s) life satisfaction (Tharp et al., 2021). On the other hand, another study concluded that caring responsibilities do not play a major role in explaining the gender gap in well-being (Adams-Prassl et al., 2020). In the present study, we attempt to reconcile these findings by exploring the moderating role of mothers’ gender ideologies (i.e., their beliefs about women’s and men’s innate abilities as parents).

## The Interaction Between Gender Ideology and Domestic Responsibilities

In line with a recent theoretical model of the effects of the COVID-19 pandemic on families (Prime et al., 2020), we argue that psychological variables such as ideologies and beliefs may play an important role for parents’ well-being during and after a crisis. We are particularly interested in how gender ideologies related to parenthood moderate the association between domestic responsibilities and women’s well-being. Ideologies can legitimize and justify inequality (e.g., Jost & Kay, 2005; Rodriguez-Bailon et al., 2017), and gender essentialism—the belief that gender differences are biologically based and therefore natural—is positively associated with support for discriminatory practices (Skewes et al., 2018). Gender essentialist ideologies in the context of parenthood reflect the degree to which individuals believe that mothers are instinctively and innately better caretakers than fathers (Gaunt, 2006). Research has shown that parents’ essentialist beliefs predict the division of childcare and housework within the home (Gaunt, 2006; Liss et al., 2013; Pinho & Gaunt, 2021), with essentialist beliefs positively predicting women’s domestic responsibilities and negatively predicting men’s share of domestic responsibilities. Essentialism has also been found to be related to parenting stress (Adams, 2020; Rizzo et al., 2013); the pressure to be a “perfect mother” is associated with higher stress levels (Henderson et al., 2016) and parental burnout (Meeussen & Van Laar, 2018). Endorsement of an essentialist ideology about mothering adds additional pressure on women to take greater responsibility for the well-being of their children (i.e., a *higher standard-higher stress* hypothesis). We therefore predict that gender essentialist beliefs about parenthood would amplify the negative effects of the increased domestic workload during the lockdown on mother’s well-being.

In contrast to this perspective, some theorists have suggested that women with more traditional ideologies are happier when enacting these traditional roles (e.g., preference theory; Hakim, 2000), so that an increased share of family responsibilities would lead to an increase in their well-being. In a critical test of this idea, a cross-national study based on the International Social Survey Programme’s (ISSP) Family 2002 dataset examined the associations between gender traditionalism and women’s happiness, life satisfaction, and stress at home (Crompton & Lyonette, 2005). Counter to the predictions of preference theory, they found that “congruent traditionalists” (i.e., women who had conservative gender attitudes and a more traditional division of domestic responsibilities) showed the *lowest* levels of well-being on all three outcome measures, highlighting the cost of essentialist beliefs about parenthood for mothers’ well-being. Other studies have found that essentialist beliefs about parenthood are associated with higher levels of stress and lower levels of life satisfaction (Rizzo et al., 2013). This is particularly true in more challenging circumstances. Homeschooling, for example, is argued to exacerbate the link between intensive mothering ideologies including gender essentialism and emotional distress because of the additional burden it places on the mother (Baker, 2019).

Thus, we predicted that mothers’ essentialist beliefs about parenthood would moderate the effect of increased domestic responsibilities during lockdown on mothers’ well-being after the lockdown, such that when mothers’ domestic workload –including childcare and housework– increases, those who strongly endorse the belief that women are essentially better caretakers will report lower well-being compared to mothers who weakly endorsed essentialist beliefs. This should be the case because mothers who strongly endorse essentialist ideologies about parenthood feel a greater responsibility for caretaking (Rizzo et al., 2013), and therefore create higher standards for themselves, which we refer to as the *higher standard-higher stress* mechanism. During a lockdown, when mothers’ share of domestic responsibilities increases because schools and daycare centers are closed, feeling the pressure to take complete responsibility for the caretaking of a child might be an unmanageable challenge for mothers. In addition, as the quality of this time is low (i.e., routine childcare vs. interactive), it is likely to be experienced as more stressful and less meaningful (Roeters & Gracia, 2016). Thus, the increased workload, together with the belief that they solely are responsible for the well-being of the child, might have severe negative consequences for these mothers’ well-being. We predict that the more a woman endorses essentialist beliefs about parenting, the more she will suffer because of an increased share of childcare and housework.

## The Norwegian Context

The present study took place in Norway. Norway is a highly gender egalitarian social-democratic welfare state (UNDP, 2019; World Economic Forum, 2020). The Norwegian social welfare policies enforce a universal childcare system, generous parental leave (including a nontransferable leave exclusive to fathers), and gender-neutral rights to leave of absence to care for sick children, supporting a work-family reconciliation for both parents and thus more equality between women and men. Over the years, these policies have contributed to a faster full-time return to paid work after childbirth for women (Rønsen & Kitterød, 2015) and resulted in a shift in collective opinion towards public childcare being the best form of care for preschool children (Ellingsæter et al., 2017). As a result, women’s and men’s employment rates in Norway are relatively comparable (in 2020 63.8% women vs. 68.8% men were in the workforce; Statistics Norway, 2021a).

Both women and men in Norway largely report gender egalitarian values regarding general societal issues (e.g., Jakobsson & Kotsadam, 2010; Kjeldstad & Lappegård, 2014). However, when it comes to matters associated with private family practices, traditional gender attitudes are still common (Kjeldstad & Lappegård, 2014; Kosakowska-Berezecka et al., 2022). Most women in Norway view themselves–and are viewed by their partners–as primarily responsible for the family’s domestic responsibilities, and women in Norway still spend almost twice as much time as men on unpaid domestic responsibilities (World Economic Forum, 2020). Although European social democratic welfare states, including Norway, show higher consistency regarding egalitarian values and practices relative to other European countries (Bühlmann et al., 2010), in a Norwegian study only 39% of dual-earner couples reported both egalitarian values and an equal division of household work (Kjeldstad & Lappegård, 2014) and almost half of Norwegian men still agree that young children will suffer if their mothers work (Lappegård et al., 2021). That is, even in the highly egalitarian Norway, practices in the home tend to reflect more traditional gender attitudes. In times of crisis, the division of domestic responsibilities overall appears to reflect traditional gender attitudes embedded in individuals, families, and the culture (for a review of influences on domestic responsibilities, see Coltrane, 2000). Research on how this affects mothers in families is therefore essential for instigating policies to moderate such setbacks in Norway, which also can inform new policies worldwide.

## The Present Research

The present study investigated the well-being of mothers of elementary school children (i.e., between 6 and 13 years) in Norway during the COVID-19 pandemic 2020 and tested which variables were related to mothers’ well-being during and after the Spring 2020 lockdown. The current study was part of a larger study that investigated the effects of the pandemic and related restrictions on parents’ and children’s well-being and attitudes towards school (Martiny et al., 2021; Thorsteinsen et al., 2021).

In Norway, public childcare, schools, and leisure activities closed on March 12th, 2020. At the same time, most non-essential businesses closed, and some people were temporarily laid off. If their jobs allowed it, people worked from home. Children under the age of twelve with two parents (or a single parent) who were essential workers (e.g., health care workers, grocery store workers) were entitled to daycare offered by the municipalities, but most children completed schoolwork from home with their parents. Children in 1^st^ to 4^th^ grade returned to school on April 27^th^, 2020. Children in 5^th^ to 7^th^ grade started to return to school between May 11^th^ and May 15^th^, 2020. By the time of the data collection (June 8^th^ to June 29^th^, 2020), the children were back in their regular classes. Other areas in Norwegian society (e.g., businesses, shops, and restaurants) also started to open up again at this time. Thus, the data of the present study were collected while society was reopening and mothers were asked to report on their well-being and share of domestic responsibilities prior to and during the lockdown retrospectively, and their current well-being post-lockdown.

In the present study, we tested two sets of hypotheses and explore the role of family-level variables. First, we focus on how the lockdown relates to mothers’ well-being. In line with previous research on mothers’ well-being (e.g., Cameron et al., 2020; Huebener et al., 2021), we tested the following hypotheses: The well-being of mothers of elementary school children would be lower during the lockdown compared to before the lockdown (H1a). As the reopening was a gradual and potentially unsettling process, we further hypothesized that mothers’ well-being during the reopening would not yet have reached their pre-lockdown level (H1b). Furthermore, based on earlier research (e.g., Adams-Prassl et al., 2020; Huebener et al., 2021), we explored the role of central family-level variables and specifically tested the links to mothers’ income, the age of the youngest child in the household, and mothers’ share of childcare and/or housework responsibilities during the lockdown. In a final step, we turned to essentialist beliefs about parenthood. We hypothesized that gender essentialism would positively predict mothers’ share of childcare and housework responsibilities independent of the COVID-19 pandemic (H2a), and that essentialism would be negatively related to mothers’ well-being during and after the lockdown (H2b), as this ideology puts additional pressure on women to bear the full responsibility for the well-being of their children. Finally, we predicted mothers’ essentialist beliefs about parenthood would moderate the effect of childcare and housework on well-being during and after the lockdown, such that the workload at home during lockdown would be associated with lower well-being for mothers at higher levels of essentialist beliefs about parenthood (H2c).

## Method

### Participants

Information about the study was sent to 266 public schools from all the regions of Norway, asking principals to distribute the invitations to parents to participate in an online questionnaire. As most parents in Norway enroll their children in public schools (95.42%; Statistics Norway, 2021b), this procedure ensured that a wide range of Norwegian parents were invited to participate. The invitation was also shared on social media (i.e., Facebook). During the data collection period (June 8^th^ to June 29^th^), 533 parents consented to participate in the study, and of these 273 participants completed the questionnaire. One participant that completed the questionnaire on July 3^rd^ was excluded. Although the study targeted both parents, most respondents were women (*n* = 230; 84.2%). We therefore decided to focus on mothers in the present analyses. Furthermore, because of our interest in mothers’ share of housework and childcare responsibilities, we only included mothers that cohabitated with a partner, giving us a final sample of 180 cohabiting mothers. The mean age of mothers in the sample was 39.96 years (*SD* = 6.11, range 23–59), they had an average of 1.98 children under age 18 (*SD* = .82, range 1–4), and the youngest child was on average seven years and nine months old (*SD* = 3.35, range five weeks to 13 years and five months). The sample included four women in a gay relationship (2.2%) and 18 of the women (10%) were not born in Norway. For 85 women (47.2%) at least one of the parents was an essential worker and in 22 of the cases (12.2%) both parents were essential workers. Nearly two-thirds (*n* = 113, 62.8%) of the sample reported a personal income between NOK 460,000 and 1,200,000 thousand, which includes the average income in Norway. Distribution of mothers’ income: NOK 0–320 thousand, (*n* = 24, 13.3%); NOK 320–460 thousand, (*n* = 39, 21.7%); NOK 460–1 200 thousand (*n* = 113, 62.8%); and 1 200–2 000 thousand, (*n* = 4, 2.2%). Average work hours were 34.33 h a week for the mother (*SD* = 13.20, range 0–74 h; 15 mothers (8.3%) reported not working at all and 40.98 h a week for their partner (*SD* = 11.70, range 0–90 h); four mothers (2.2%) had partners who did not work. In general, women perceived the spread of the coronavirus in their municipality to be low (*M* = 1.27, *SD* = .88, on a scale from 0 = *low infection rate* to 4 = *very high infection rate*) which accurately reflected the reality in most Norwegian municipalities. The study was approved by the Norwegian Center for Research Data and the board for research ethics at the Department of Psychology, UiT The Arctic University of Norway before data collection.

### Statistical Power

We conducted sensitivity analyses with G*Power to compute the potential effect size that could be detected given the alpha, power, and sample size. For the repeated measures ANOVA, with the current sample (*N* = 180) and three measurements, we were able to detect an effect size of f^2^ = .17 (small effect) with a power of .95. For the linear multiple regressions, with the current sample (*N* = 180) and three predictors, we were able to detect an effect size of f^2^ = .06 (small effect) with a power of .95.

### Measures and Procedure

In the questionnaire, participants were first asked to give informed consent. They were then asked the set of questions about their life before the COVID-19 pandemic, followed by questions related to the lockdown (when schools were closed), and the present situation (after reopening). Due to the time it took to obtain ethical consent, we were unable to start data collection earlier. A retrospective approach was therefore applied for pre-measures and lockdown measures. Items within each measure were presented in a randomized order. At the end of the questionnaire, participants had the opportunity to provide their e-mail address to take part in a lottery for five gift cards with a value of NOK 500.

#### Well-Being

Well-being was measured with the 5-item World Health Organization Index (Topp et al., 2015). The index contains positively phrased items, e.g., “I have felt cheerful and in good spirits,” scored from 0 (*none of the time*) to 5 (*all of the time*). In line with past research, the raw sum score (with a range from 0 to 25) was multiplied by four so that the final score had a possible range from 0 (*absence of well-being*) to 100 (*maximum well-being*). We adapted the instructions so that participants were first asked to recall their well-being before the outbreak of COVID-19, i.e., “Think back to how you felt *before* the society closed down” (α = .89), then during the lockdown of society, i.e., “Think back to how you felt *while* schools were closed” (α = .90), and then after the reopening of the society, i.e., “Please indicate how you felt *after* the schools reopened” (α = .90).

#### Essentialist Beliefs About Parenthood

Essentialist beliefs about parenthood were measured by seven items designed to assess essentialist perceptions of men and women as parents (Gaunt, 2006). Items were scored from 1 (*strongly disagree*) to 5 (*strongly agree*), e.g., “Fathers have to learn what mothers are able to do naturally” and “Maternal instincts enable mothers to identify baby’s needs”. Responses were coded so that a higher score reflects stronger essentialist beliefs about men and women as parents (α = .84).

#### Domestic Responsibilities

Domestic responsibilities were measured with nine items developed for the current study. In the questionnaire, participants were asked about the distribution of domestic responsibilities between themselves and their partner twice: first regarding the time before the outbreak of COVID-19, and then during the lockdown. For both time points, exploratory factor analyses of the nine domestic responsibilities items indicated that they loaded on two separate factors (factor 1: housework, factor 2: childcare) with eigenvalues above one. Factor loadings and communalities are reported in Table 1. The correlations between the factors were *r*_before_ = .39 and *r*_lockdown_ = .56. A composite score was created for the four items loading on the factor measuring housework responsibilities, e.g., “How much of the dishes at your house do you/your partner do, respectively?”, and the five items loading on the factor measuring childcare responsibilities, e.g., “How much of the childcare of caring for your kids at home do you/your partner do, respectively?” In addition, the childcare subscale included one item asking who was responsible for bringing the child to school. This item was excluded to make the before and during lockdown childcare responsibility subscales comparable. Respondents answered on a scale from 1 (*my partner does it all*) to 7 (*I do it all*). Participants first answered the domestic responsibilities measures about the situation before COVID-19 (housework α = .62, childcare α = .82) and then again during the lockdown (housework α = .72, childcare α = .88).

**Table 1:** Factor Loadings and Communalities for Promax Rotated Two-Factor Solution for Nine Domestic Responsibilities Items (*N* = 180)

	Factor		
	Child care	House work	h^2^
**Before the lockdown**			
Who does the childcare by spending time with the children?	**.82**	–.03	.65
Who does the childcare by fulfilling the children’s emotional needs?	**.79**	–.17	.56
Who does the childcare by taking care of the children at home?	**.73**	.08	.59
Who does the childcare by fulfilling their physical needs?	**.70**	.06	.53
Who helps the children with their homework?	**.44**	.17	.28
Who does the dishes?	–.04	**.69**	.46
Who does the cleaning?	.11	**.60**	.41
Who cooks?	–.07	**.57**	.30
Who does the laundry?	.05	**.34**	.13
**During the lockdown**			
Who did the childcare by taking care of the children at home?	**.84**	.01	.71
Who did the childcare by spending time with the children?	**.83**	.06	.76
Who did the childcare by fulfilling the children’s emotional needs?	**.81**	–.13	.55
Who did the childcare by fulfilling their physical needs?	**.75**	.07	.63
Who helped the children with their homework?	**.64**	.03	.44
Who did the dishes?	–.09	**.81**	.58
Who did the cleaning?	–.02	**.68**	.45
Who cooked?	–.19	**.52**	.42
Who did the laundry?	.04	**.44**	.22

Extraction method: Principal Axis Factoring; Rotation method: Promax with Kaiser normalization; Rotation converged in 3 iterations

In addition to the variables reported in this paper, the parent questionnaire contained the following measures: changes at the children’s school, general experience, perception of child’s well-being before, during, and after lockdown, perception of children’s attitudes towards school before and after lockdown, attitudes towards gender equality, and demographics about themselves, their child, and their partner. At the end, participants were given the opportunity to provide additional information or comments. As part of a larger project, we also asked their children to fill in a children’s questionnaire. Results from the children’s questionnaire are not reported in the present paper.

## Results

Table 2 includes descriptive statistics and correlations for measures included in the analyses. Data and SPSS syntax can be found online on the Open Science Frame: OSF.IO/4FRK2. Missing data were handled using pairwise deletion and errors were checked for non-normality before analyses.

**Table 2:** Descriptive Statistics and Correlations for Analyses Variables

Variable	*N*	*M*	*SD*	1	2	3	4	5	6	7	8	9	10
1. Pre-lockdown wellbeing	180	63.80	19.35	-									
2. Lockdown wellbeing	180	59.67	21.07	.33^**^	-								
3. Reopening wellbeing	180	61.44	19.25	.70^***^	.54^***^	-							
4. Gender essentialist beliefs	180	2.38	0.77	–.04	–.09	–.13	-						
5. Pre-lockdown childcare	179	4.80	.80	–.13	–.01	–.13	.23^**^	-					
6. Lockdown childcare	180	4.97	1.03	–.16^*^	–.09	–.16^*^	.24^**^	.69^***^	-				
7. Pre-lockdown housework	180	4.95	0.97	–.00	–.13	.02	.16^*^	.31^***^	.32^***^	-			
8. Lockdown housework	179	5.00	1.07	–.04	–.15	–.01	.22^**^	.31^**^	.49^***^	.84^***^	-		
9. Income	180	2.54	0.75	.18^*^	–.11	.09	–.15^*^	–.16^*^	–.11	–.10	–.06	-	
10. Age, youngest child	180	7.76	3.35	.23^**^	.21^**^	.27^***^	–.07	.08	–.01	–.05	–.03	–.04	-

Values reflect ^*^p < .05; ^*^p < .01; ^***^p < .001

### Changes in Well-being

First, we tested whether mothers’ well-being decreased during the lockdown as compared to before the lockdown (H1a), and whether their well-being during the reopening had recovered to the level before the lockdown (H1b). A repeated measures ANOVA with a Huyn-Feldt correction determined that mothers’ well-being differed between the three measurement points, *F*(1.63, 291.44) = 4.05, *p* = .026, η^2^p = .02. Pairwise comparisons of means showed significant reduction in mothers’ well-being during the lockdown (*M* = 59.67, *SD* = 21.07) compared to before the lockdown (*M* = 63.80, *SD* = 19.35, *t*(179) = –2.37, *p* = .019). Well-being after reopening (*M* = 61.44, *SD* = 19.25) did not significantly differ from well-being during lockdown (*t*(179) = 1.23, *p* = .220) and was significantly lower than well-being before the lockdown (*t*(179) = –2.11, *p* = .036). The findings were consistent with the hypotheses.

Second, we conducted exploratory regression analyses for family-level variables that potentially can help account for the reduction in well-being of mothers (during the lockdown and after reopening, controlling for pre-lockdown well-being). Of the family-level variables included in our study, only income was negatively related to lockdown well-being and age of youngest child was positively related to lockdown well-being. Reopening well-being was positively associated with one family-level variable, namely age of youngest child (positive). Additionally, we observed a non-significant, negative association for housework during lockdown predicting lockdown well-being. All the family-level variables tested are presented in Table 3. Because of the significant association between well-being and income and age of youngest child, we control for these two variables in all subsequent analyses for well-being.

**Table 3:** Summary of Regression Results for Family-Level Variables as Predictors of Well-Being During Lockdown and Well-Being at Reopening

	Lockdown well-being				Reopening well-being			
	b*	*t*	df	*p*	b*	*t*	df	*p*
Age	.07	1.00	178	.321	.05	.857	178	.392
Age of youngest child	.14	2.00	179	.047	.11	2.09	179	.038
Belonging to risk group	–.05	0.70	179	.482	–.10	–0.73	179	.466
Essential workers	–.07	–0.92	179	.358	–.06	–1.12	179	.263
Income	–.17	–2.45	179	.015	–.04	–0.80	179	.427
Lockdown childcare	–.04	–0.53	179	.600	–.05	–0.92	179	.359
Lockdown housework	–.13	–1.88	178	.062	.02	0.45	178	.656
Norwegian*	–.08	–1.07	179	.285	.01	0.14	179	.891
Number of children	–.05	–0.62	179	.532	–.02	–0.35	179	.728
Perceived infection rate	.02	0.31	179	.753	–.02	0.36	179	.719
Work hours	–.08	–1.07	176	.288	–.02	–0.28	176	.783
Work hours of partner	–.11	–1.58	177	.116	–.04	–0.75	177	.453

All regressions include pre-lockdown well-being as a control. Variables are entered alphabetically. Lockdown well-being and Reopening well-being was measured by WHO-5; Belonging to risk group is a dummy variable indicating if = 1, or if not = 0, either child or parent (or both) belong to COVID-19 risk group; Essential workers is a dummy variable indicating if = 1, or if not = 0, either parent is employed in a critical occupation; Born outside Norway is a dummy variable indicating if = 0, or if not = 1, the participant was born in Norway

b* = standardized regression coefficient

### Role of Gender Essentialist Beliefs About Parenthood

In line with H2a, gender essentialist beliefs about parenthood predicted mothers’ responsibility for childcare before the lockdown, *b* = .23, *t*(178) = 3.10, *p* = .004. Gender essentialist beliefs also predicted mothers’ responsibility for childcare during the lockdown, *b* = .24, *t*(179) = 3.25, *p* = .001. In addition, a repeated measures ANOVA showed that mothers perceived more childcare responsibilities during the lockdown as compared to before the lockdown, *F*(1, 178) = 9.46, *p* = .002, η^2^p = .05. Gender essentialist beliefs about parenthood predicted mothers’ responsibility for housework before the lockdown, *b* = .16, *t*(179) = 2.21, *p* = .029, and during the lockdown *b* = .22, *t*(178) = 2.93, *p* = .004. A repeated measures ANOVA showed no change in mothers’ share of housework responsibilities between before and during the lockdown, *F*(1, 178) = 1.83, *p* = .178.

In line with H2b, gender essentialist beliefs did not predict well-being before the lockdown, *b* = .01, *t*(179) = .11, *p* = .878, or during the lockdown, *b* = –.10, *t*(179) = –1.40, *p* = .164 (controlling for pre-lockdown well-being and the family-level controls); however gender essentialist beliefs negatively predicted well-being after the reopening of society, *b* = –.11, *t*(179) = –2.03, *p* = .044. Endorsing gender essentialist beliefs about parenting were associated with lower levels of well-being post-lockdown.

### Gender Essentialist Beliefs as a Moderator of Domestic Responsibilities–Well-Being Link

**Fig. 1:**
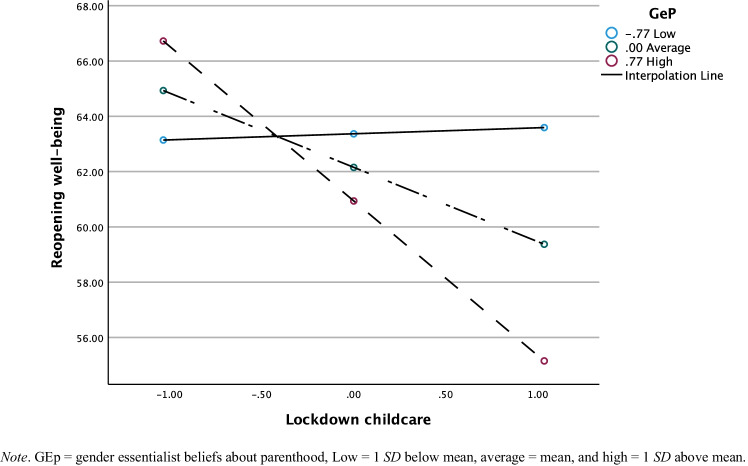
Moderating Effect of Gender Essentialist Beliefs About Parenthood on Association between Childcare Responsibilities During Lockdown and Well-Being at Reopening

Finally, we tested H2c, which predicted that gender essentialist beliefs about parenthood would moderate the effect of domestic responsibilities during the lockdown on mothers’ well-being after reopening, using Hayes (2013) PROCESS macro for SPSS. We selected well-being at reopening as the outcome variable to test the moderation analyses for gender essentialist beliefs and domestic responsibilities (i.e., childcare and housework) during the lockdown to ensure temporal precedence. In line with our hypothesis, gender essentialist beliefs moderated the effect of lockdown childcare on well-being for women, *F*(5, 174) = 5.37, *p* < .001 (regression coefficients are presented in Table 4; see Fig. 1). For women with low gender essentialist beliefs, there was no association between childcare responsibilities during lockdown and well-being at reopening, *b* = .23, *t*(174) = .124, *p* = .902. For women with average gender essentialist beliefs, there was a non-significant negative association between greater share of childcare and reduced well-being, *b* = –2.64, *t*(174) = –1.94, *p* = .054. For women with high gender essentialist beliefs, greater childcare responsibilities during lockdown was associated with lower well-being at reopening, *b* = –5.50, *t*(174) = –2.92, *p* = .004, indicating that perceiving oneself as responsible for a greater share of childcare during the lockdown was particularly costly for the well-being of mothers with higher gender essentialist beliefs about parenthood. The increase in explained variance in well-being at reopening due to the interaction was Δ*R*^2^ = .03, *F*(1, 174) = 5.04 *p* = .026 for childcare. The pattern of results remained unchanged when we controlled for pre-lockdown well-being.

**Table 4:** Summary of Regression Analyses for Well-Being at Reopening as a Function of Gender Essentialist Beliefs and Domestic Responsibilities

	Childcare			Housework		
Independent variables	*B*	*t*	*p*	*B*	*t*	*p*
Constant	45.41 [33.70; 57.12]	7.65	< .001	45.33 [33.51; 57.16]	7.57	< .001
Age youngest child	1.50 [.70; 2.31]	3.69	< .001	1.48 [.67; 2.30]	3.59	< .001
Income	1.99 [–1.64; 5.63]	1.08	.280	2.06 [–1.59; 5.72]	1.11	.268
Domestic responsibilities	–2.64 [–5.31;.41]	–1.94	.054	0.47 [–2.12; 3.05]	0.36	.721
Gep	–.83 [-4.52; 2.86]	–.44	.658	–2.28 [–5.92; 1.36]	–1.24	.217
Domestic responsibilities*GeP	–3.70 [–6.95; –.45]	2.25	.026	–3.89 [–7.05; –.74]	–2.44	.016

*GEp* gender essentialist beliefs related to parenthood

**Fig. 2:**
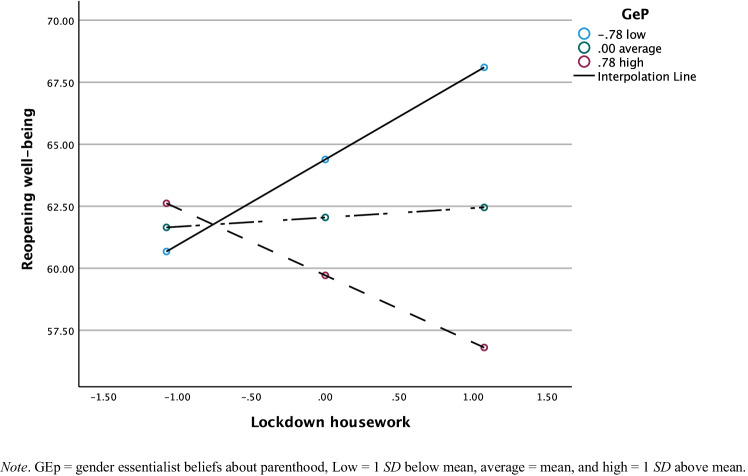
Moderating Effect of Gender Essentialist Beliefs About Parenthood on Association between Housework Responsibilities During Lockdown and Well-Being at Reopening

For housework, gender essentialist beliefs moderated the association between housework responsibilities during lockdown and well-being at reopening, *F*(5, 173) = 4.69, *p* = .001 (regression coefficients are presented in Table 4; see Fig. 2). However, there were no significant conditional effects of perceived share in housework on reopening wellbeing at different levels of gender essentialist beliefs about parenthood. For mothers with low gender essentialist beliefs, the association between housework and reopening well-being was, *b* = 3.49, *t*(173) = 1.95, *p* = .052. For mothers with average gender essentialist beliefs, the association between housework and reopening well-being was, *b* = .47, *t*(173) = .36, *p* = .721. For mothers with high gender essentialist beliefs, the association between housework and reopening well-being was, *b* = –2.56, *t*(173) = –1.41, *p* = .162. The increase in explained variance in reopening well-being due to the interaction was Δ*R*^2^ = .03, *F*(1, 173) = 5.95, *p* = .016 for housework. The interaction between housework and reopening wellbeing no longer reached the conventional significance level, *b* = –2.08, *t*(172) = –1.75, *p* = .083 when we controlled for pre-lockdown well-being.

## Discussion

The present work adds to the emerging literature on the well-being of mothers with school-aged children during the COVID-19 pandemic. Results of the present study show that mothers reported lower well-being both during the lockdown and after reopening compared to their well-being before the lockdown. There were no differences in mothers’ wellbeing during the lockdown and after reopening. In addition, in line with earlier research (Adams-Prassl et al., 2020; Huebener et al., 2021), we found that mothers’ well-being was related to family-level variables during the crisis, including income and age of youngest child.

In addition, the present study showed that gender essentialist beliefs about parenthood were related to mothers’ share of childcare and housework before and during the lockdown. Mothers who believed women have an innate advantage in parenting perceived themselves as having taken on more responsibilities in the home, and these essentialist beliefs about parenthood were also negatively related to their well-being during and after the lockdown. In line with our predictions, we found that endorsement of essentialism was negatively related to well-being during the lockdown, but not to well-being before the lockdown. In addition, we found that essentialist beliefs about parenting moderated the association between mothers’ perceived share of childcare and housework during the lockdown and mothers’ well-being at reopening. This means that gender essentialist beliefs about parenting were particularly detrimental to women’s well-being as their perceived share of domestic responsibilities during the lockdown increased. We theorize that this is the case because mothers who strongly endorse gender essentialist beliefs set a high standard for themselves in their parenting (i.e., as the only one truly equipped to meet their children’s needs), which becomes increasingly hard to achieve in times of crisis when parents need to juggle work, homeschooling, housework, and parenting. We argue that the belief that mothers are the more essential and effective parent creates additional stress and sense of responsibility (Adams, 2020; Baker, 2019; Rizzo et al., 2013), and therefore reduces mothers’ well-being. Future research should explicitly test this *high standards-high stress* explanation for the negative relation between gender essentialism and mothers’ well-being in challenging situations. However, regardless of the specific mechanism underlying this relation, our findings demonstrate that family-level variables (such as perceived changes in responsibility for domestic tasks) interact with individuals’ ideologies in affecting mental health. As outlined earlier, conflicting results have been reported in research examining whether the increase in domestic responsibilities that women experienced during the lockdown could explain the gender gap in well-being. Measuring individuals’ gender ideologies, such as essentialist beliefs about parenthood, might help to explain these inconsistent findings.

The results also demonstrated that a large increase in mothers’ childcare responsibilities during the lockdown were particularly harmful for mothers who highly endorsed essentialist gender ideologies. We found a similar, but non-significant pattern for the moderating role of endorsement of essentialism on the association between these perceived increases in housework and mothers’ well-being, where the overall interaction was significant but not the conditional effects. However, when controlling for mothers’ well-being before the lockdown, the moderating effect of essentialist beliefs about parenthood was no longer significant. We see two potential reasons for the lack of an effect with housework responsibilities: First, our analyses show that whereas the amount of childcare significantly increased during the lockdown due to closed childcare centers and schools, housework might have increased only slightly. In addition, our measurement of essentialist ideologies targeted childcare (e.g., “Fathers have to learn what mothers are able to do naturally”). Although we expected similar effects for both domains, the childcare-focused measure of essentialism in parenting may explain the stronger effects for childcare as compared to housework in our study.

When interpreting the present findings, one needs to keep in mind that Norway was one of the countries that was not hit particularly hard by the first wave of the COVID-19 pandemic. By the end of our data collection, 936 people had been admitted to the hospital and 250 people had died due to the coronavirus in Norway (cumulative numbers up to June 28^th^ 2020; Norwegian Institute of Public Health, 2020). At the first peak of the crisis in Spring 2020 (March and April 2020), the numbers of daily infections were relatively low (around 300 per day) and most hospitals did not reach capacity. In addition, there were large parts of Norway that reported very few (if any) infections. For this reason, it seems likely that the decrease in mothers’ well-being would be much stronger in countries that suffered more in the early months of the pandemic (e.g., Italy, Spain, U.S.). Thus, we expect that in comparison to the situation in many other countries, the present results underestimate the effect of the pandemic on mothers’ well-being. However, in other ways Norway is a particularly interesting context for studying the impact of the pandemic on mothers’ well-being. Earlier research found that generous family policies, as provided in Norway, are associated with smaller differences in well-being between parents and non-parents (Glass et al., 2016; Nordenmark, 2021). The present study highlights, however, that this increased equality in Norway might still be vulnerable and easily disrupted when structural variables (such as the shutdown of the educational system) change.

### Limitations and Future Research Directions

Despite the important contribution that the present work makes to our knowledge of how the COVID-19 pandemic and related restrictions affected mothers of school-aged children, some limitations of the present study need to be discussed. First, all measures were assessed at the same time point, namely during June when the reopening of the Norwegian society was well on its way. Therefore, both the pre-lockdown measures and the lockdown measures may be affected by recall biases. However, past research suggests that European Americans tend to exaggerate their well-being in retrospective judgments, due to a nonconscious tendency to focus on positive information (Oishi, 2002). This bias should work against the lower levels of well-being we found in our retrospective measures during the lockdown. Still, the lockdown was a distinct and impactful time in people’s lives and might therefore be remembered more vividly (see Brown, 2021).

In line with past findings that have shown that essentialist ideologies (i.e., as a part of intensive parenting) exacerbate stressful parenting environments (e.g., homeschooling; Baker, 2019), we predicted they would also moderate the association between domestic work and well-being. The present findings supported our hypotheses in the context of childcare responsibilities. At the same time, the correlational nature of our work prohibits us from drawing any conclusions about causal relations between the variables of interest. However, we do not see a logical argument for reversed causal patterns (i.e., a reduction in well-being causing more essentialist beliefs or domestic responsibilities). In addition, we have only focused on one moderator, and there may be other moderators of this association to be explored by future research, such as mothers’ positive emotions and trait resilience in the face of adversity (e.g., in response to the September 11^th^ terrorist attacks; Fredrickson et al., 2003).

A final limitation that we need to mention is that self-selection might have played a role. As described in the methods, most children in Norway attend public schools, regardless of parents’ socioeconomic status. Thus, we are optimistic that the invitation to participate in the study was received by a relatively representative group of Norwegian parents. At the same time, the parents who were willing to follow the invitation and respond to our questionnaire could have differed from the general population; for example, mothers who volunteered may have had more time and energy to fill in the questionnaire. However, it might also be the case that parents who were suffering during the pandemic were more motivated to share their experience, and therefore more willing to complete the questionnaire. In a recent study by Sischka et al. (2020) using the same well-being measure, a representative Norwegian sample reported a mean score of 71.56 before the pandemic. This data favors the latter explanation, as the mothers in our sample had a mean score of 59.67 during the lockdown. Nevertheless, as Sischka et al.’s sample included both men and women (49.4%), and only working adults, these two opposing predictions still make it hard for us to conclude how self-selection might have influenced our results. It is possible that both groups of parents were included in the present sample and thus the present results might to some degree be representative of the situation of mothers in Norway during the COVID-19 pandemic.

### Practice Implications

The present study highlights the importance of addressing not only external factors that disproportionately burden mothers in times of crisis (e.g., childcare, income loss) but also the ideologies and cultural values that put additional pressure on women to take on a greater share of childcare and housework, and to hold themselves to a higher standard for this work. Parental leave may be one practical way to enact these changes on a global level. Increasing the amount of parental leave fathers take relative to mothers has been found to produce a more equal distribution of caretaking and household responsibilities (Meil, 2013; Patnaik, 2019), and Omidakhsh et al. (2020) have shown that paternal leave policies lead to more egalitarian gender norms and attitudes. In addition, parent education programs should tackle harmful gender ideologies and encourage fathers’ involvement (e.g., by including fathers in perinatal parenting classes; Lee et al., 2018), and parenting magazines should highlight the negative aspects of essentialism for mothers’ well-being (Greve Spees & Zimmerman, 2003). As the present study suggests, this concept may be especially important in times of stress or crisis, and policymakers should take these factors into account when making decisions about how to handle similar health-related crises in the future.

### Conclusion

The present work investigated changes in the well-being of mothers with elementary school children in Norway around the COVID-19 lockdown. Results showed that mothers’ well-being decreased during the lockdown and that this decrease was related to family-level variables as well as mothers’ essentialist beliefs about parenthood. Importantly, mothers who strongly believed that women were instinctively and innately better caretakers than fathers reported lower well-being in relation to the greater childcare and housework responsibilities that occurred during the lockdown of the Norwegian society. Beyond this specific context, the present study demonstrates how essentialist gender ideologies may exacerbate existing inequities in well-being in challenging times.
